# Homeostatic neuro-metasurfaces for dynamic wireless channel management

**DOI:** 10.1126/sciadv.abn7905

**Published:** 2022-07-06

**Authors:** Zhixiang Fan, Chao Qian, Yuetian Jia, Zhedong Wang, Yinzhang Ding, Dengpan Wang, Longwei Tian, Erping Li, Tong Cai, Bin Zheng, Ido Kaminer, Hongsheng Chen

**Affiliations:** 1 Interdisciplinary Center for Quantum Information, State Key Laboratory of Modern Optical Instrumentation, ZJU-UIUC Institute, Zhejiang University, Hangzhou 310027, China.; 2 ZJU-Hangzhou Global Science and Technology Innovation Center, Key Laboratory of Advanced Micro/Nano Electronic Devices and Smart Systems of Zhejiang, Zhejiang University, Hangzhou 310027, China.; 3 Jinhua Institute of Zhejiang University, Zhejiang University, Jinhua 321099, China.; 4 DAMO Academy, Alibaba Group, Hangzhou 310025, China.; 5 Air and Missile Defense College, Air Force Engineering University, Xi’ an 710051, China.; 6 Shanghai Key Laboratory of Navigation and Location-based Services, Shanghai Jiao Tong University, Shanghai 200240, China.; 7 Department of Electrical and Computer Engineering, Technion—Israel Institute of Technology, Haifa 32000, Israel.

## Abstract

The physical basis of a smart city, the wireless channel, plays an important role in coordinating functions across a variety of systems and disordered environments, with numerous applications in wireless communication. However, conventional wireless channel typically necessitates high-complexity and energy-consuming hardware, and it is hindered by lengthy and iterative optimization strategies. Here, we introduce the concept of homeostatic neuro-metasurfaces to automatically and monolithically manage wireless channel in dynamics. These neuro-metasurfaces relieve the heavy reliance on traditional radio frequency components and embrace two iconic traits: They require no iterative computation and no human participation. In doing so, we develop a flexible deep learning paradigm for the global inverse design of large-scale metasurfaces, reaching an accuracy greater than 90%. In a full perception-decision-action experiment, our concept is demonstrated through a preliminary proof-of-concept verification and an on-demand wireless channel management. Our work provides a key advance for the next generation of electromagnetic smart cities.

## Teaser

Homeostatic neuro-metasurfaces are demonstrated to automatically and monolithically manage wireless channel in dynamic.

## INTRODUCTION

**Fig. 1.**
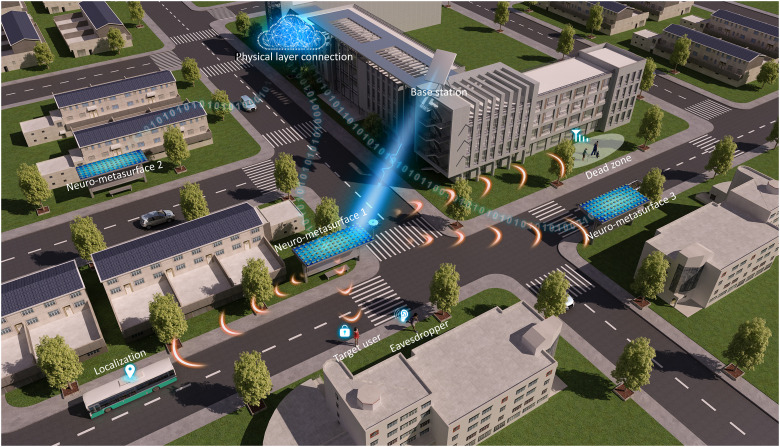
Illustrative scenario of homeostatic/self-acting neuro-metasurfaces in an EM smart city and infrastructures. A homeostatic neuro-metasurface mounted on the bus stop monolithically manages the wireless channel in a disordered and dynamic environment. A myriad of scenarios can thus be envisioned, such as compensating for signal loss, localizing a moving target, and extending signal coverage in a dead zone. Various homeostatic neuro-metasurfaces can also team up for collaborative work or communicate with third parties via physical layer connection. This scheme reduces the energy consumption and hardware cost over conventional setup, such as oscillators, mixers, and amplifiers, and thus enables a large advance toward flexible and efficient IoT devices.

Smart city is a generic term used to describe an urban area that leverages information and communication technologies to optimize transportation systems, social sustainability, resource allocation, and other community services (*1*). In particular, recent decades have witnessed an unprecedented promotion because of the big advances in the Internet of Things (IoT) and artificial intelligence (*2*). Much effort has been inaugurated to pursue intellectualization at the data link level and network level (*3*–*4*). However, the physical level, the wireless channel, an electromagnetic (EM) link between the transmitter and the receiver with complex propagations inside, also plays an important role (*5*). As conceptualized in Fig. 1, if we can manage the wireless channel as desired, then a radically new EM smart city/infrastructure could be created (*6*). In such a vision, many intriguing applications will be facilitated. For example, one can physically cancel off the signal sent from the base station at the location of an eavesdropper, thus effectively reducing information leakage.

A fundamental backbone to create the aforementioned vision is to physically manage the wireless channel and modify the EM environment even in a disordered surrounding (*7*). Conventionally, the engineering of wireless channel typically necessitates high-complexity and energy-consuming hardware at base stations, and it is limited by lengthy and iterative optimization strategies (*8*–*9*). In this context, densely deploying wireless relay techniques with signal regeneration and retransmission will entail intensifying hardware expenditures and maintenance costs. In the past years, metasurfaces, an artificial wave-functional interface composed of arrays of subwavelength resonators, have attracted extensive attention for manipulating wireless channels in a green and cost-effective manner (*10*–*11*). By introducing spatiotemporally varying optical response into metasurfaces, we can arbitrarily shape wavefront and adjust polarization state of the impinging waves. Thus far, metasurfaces are being anticipated to bring a new twist in fifth-generation (5G) wireless communication (*12*), intelligent reflection surface (*13*), imaging recognition (*14*), and beyond (*15*–*17*).

However, the related metasurface-based works share a common limitation: either they are static in nature (set in stone after fabrication) or work in a trial-and-error mode to satisfy user demands (dependent and iterative) (*18*). If one wants to overcome this, then a necessary step is to quickly unlock and streamline the intricate interactions among metasurfaces, dynamic environment, and user demands. Deep learning, as a powerful data-driven method, has recently been welcomed to expedite the on-demand design of metamaterials (*19*–*27*) and photonic crystals (*28*–*30*). The state-of-the-art works can be divided into two categories: accurately encapsulate optical responses for a given structure (forward prediction) and inversely design physical structures for a given optical response (inverse design) (*31*–*32*). Compared with full-wave numerical simulations and physics-based approaches, deep learning has found to be efficient, time-saving, and reusable because it is able to unearth obscure optical characteristics and latent physics from a suitable amount of data (*33*). For wireless channel management, however, the related deep learning works may become inefficient and even invalid because of the following reasons. First, these works are mostly limited to subwavelength and individual elements (local design, ignoring the coupling effects among adjacent elements), while for large-scale metasurfaces, the streamline design is in high demand but still elusive (global design). Second, a majority of works only focus on the analytical design of passive elements, while the reconfigurable explorations in experiment are scarce, let alone to an unmanned experiment (*34*).

To overcome these limitations, we propose the concept of homeostatic/self-acting neuro-metasurfaces to globally manage wireless channel during the propagation process and automatically cater to user demands in dynamic environment. Homeostatic neuro-metasurfaces are a deep learning–driven planar array consisting of a large number of active elements; each active element imparts an independent amplitude/phase/polarization modulation to the incident waves. In this work, we consider a mechanical-actuating neuro-metasurface, and for each neuro-element, the reflection phase is separately tuned by mechanical rotation. With a moderate number of data and associated data augmentation technique, a generative model for global inverse design is developed. Compared with conventional adaptive strategies, the homeostatic neuro-metasurfaces involve only one single-forward computation, thus saving a notable amount of computational time (*35*). In experiments, we build up a complete perception-decision-action system to mimic real-world scenarios and carry out two progressive experiments: A proof-of-concept experiment to verify the global inverse design model and an on-demand wireless channel management experiment. Our work opens a new avenue for the next generation of EM smart infrastructure and, more generally, pushes metasurfaces to a new horizon, empowering inanimate metasurfaces with human wisdom (*9*, *36*).

## RESULTS

### Neuro-metasurfaces enabled EM smart cities and infrastructures

The applications of neuro-metasurfaces are abundant with the merits of low profile, light weight, and conformal geometry. As an epitome of EM smart cities, Fig. 1 depicts a vision of future bus stop, where homeostatic neuro-metasurfaces are mounted on a shed to holistically humanize the wireless channel. Several typical scenarios are illustrated. For example, in a dead zone (the direct link between users and the base station is blocked), neuro-metasurfaces can be leveraged as transfer stations to create a virtual line-of-sight link. For directional signal propagation, neuro-metasurfaces can adaptively adjust each element to strengthen the desired signal or suppress the undesired signal. For wireless communication, one can encode information into spatiotemporal sequences to tune neuro-metasurfaces in both time and space (space- and frequency-division multiplexing) (*12*). Furthermore, multiple neuro-metasurfaces and even third-party devices can be interfaced through physical layer connection for collaborative works. In these examples, neuro-metasurfaces can be flexibly deployed in wireless networks and relieve the heavy reliance on traditional radio frequency components, making a big step toward low-cost and green IoT devices.

To reach this goal, the neuro-metasurface architecture should include three key components: perception, decision-making, and action. We mimic them by EM detector, deep learning algorithm, and reconfigurable metasurfaces, respectively. For most of the metasurface-related devices, they typically work for a specific EM mode that is assumed to be a priori known (*37*–*38*). However, in practice, these factors may vary all the time, making a predesigned metasurface device inefficient. We treat these factors (acquired by EM detector) as input parameters of a deep learning algorithm, together with a customer-defined wireless channel. According to the two sets of inputs, the deep learning algorithm will quickly generate candidate metasurface patterns. Although programmable and reconfigurable metasurfaces have been extensively studied and underpin the reconfigurability of some established technologies, they also need to work in tandem with outside helps and in a trial-and-error mode for a customer-specific requirement.

### Global inverse design of homeostatic neuro-metasurfaces

For the core of the neuro-metasurface architecture, we consider deep learning algorithm to bridge wireless channel to large-scale neuro-metasurfaces. This remains out of reach for the existing inverse design works because they are mostly limited to individual subwavelength elements/unit cells (*39*). These works buildup a bidirectional channel between physical structures and their local optical properties. However, they inherently neglect the nonlinear interactions and elusive couplings between adjacent structures because of the imperfect theoretical assumption (*40*–*41*), and for large-scale metasurfaces, the number of degrees of freedom increases. The seemingly simple increase will make the whole solution space expand exponentially, leading to a burdensome task on dataset collection and algorithm modeling. This thorny issue also widely exists in other numerous applications, such as multidimensional physical crystals and multipixel holograms (*42*–*43*).

**Fig. 2.**
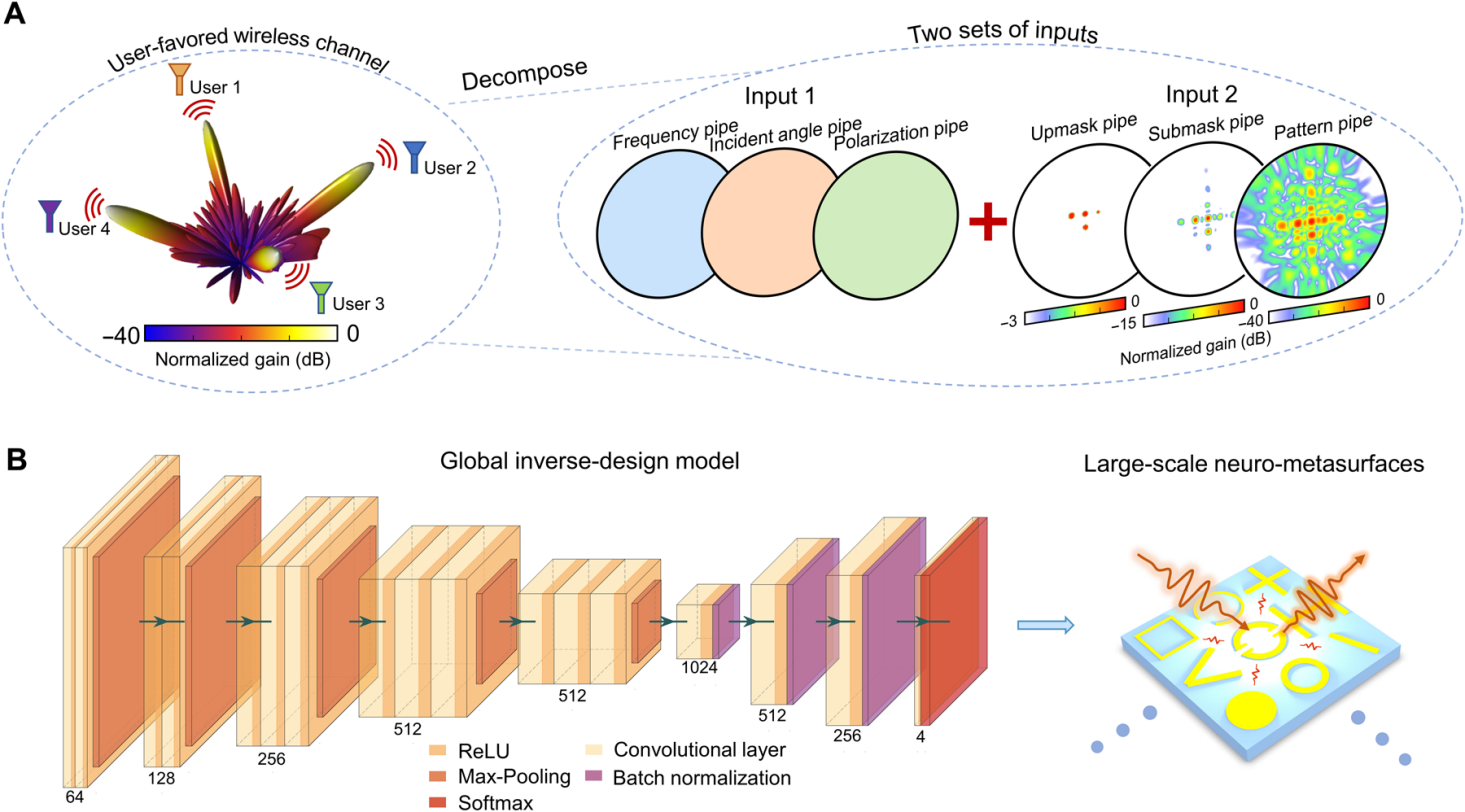
Global inverse design of homeostatic neuro-metasurfaces. (**A**) Data preprocessing. The input wireless channel is decomposed into two sets of inputs including input 1 (frequency, incident angle, and polarization pipes) and input 2 (upmask, submask, and pattern pipes) to effectively extract signal characteristics. All pipes are expressed in polar coordinates. The upmask and submask pipes represent the 3- and 15-dB down boundaries of the pattern channel, respectively. (**B**) Global inverse design model. The input is a user-favored wireless channel, and the output is the reconfiguration of homeostatic neuro-metasurface. The encoder-decoder structure is composed of convolutional layers, transposed convolutional layers, pooling layers, batch normalization layers, and so on; see note S3 for a detailed description.

To tackle the above challenges, the pipeline of data preprocessing and the generative network structure are proposed in Fig. 2. First, we directly consider full-wave simulation data or experimental measured data of neuro-metasurfaces as training data; this way, the complicated interaction between adjacent structures is involved. Then, we decompose each radiation pattern (expressed in polar coordinates) into three feature extraction pipes (pattern, upmask, and submask pipes) and set the frequency, incidence, and polarization of incident waves as the other set, each of which has a dimension of 200 × 200 (Fig. 2A). The pattern pipe is the raw radiation pattern from a simulation or experiment. The upmask and submask pipes represent the 3- and 15-dB down boundaries of the pattern pipe, which represent the half power beam width of the radiation pattern and the radiation pattern lobes and side lobe levels, respectively. Compared with traditional methods that take a data matrix as input directly, the representation of polar coordinates and three feature extraction pipes not only preserves the inherent spatial association information but also preprocesses the EM features effectively. In this manner, the characteristics of the radiation pattern can be easily extracted with fast convergence (*44*).

Regarding the network architecture, a typical encoder-decoder structure comprising convolutional layers, pooling layers, and batch normalization layers is designed. Owing to its hierarchical structure, such a network structure promises rapid inference, strong generalization, translation, and scale invariance (*45*), making it a superior candidate. For a specific task, the performance of accuracy and the decline of loss are comprehensively considered to determine the encoder-decoder architecture. To facilitate the training process, we identify two evaluation indices, i.e., the cross-entropy loss and accuracy rate (*46*), to minimize the inconsistences of the 20 × 20 neuro-metasurface profiles in the ground truth and predicted cases with a labeled state 0/1/2/3; see note S3 for the details of the neural network.

### Experimental buildup of homeostatic neuro-metasurfaces

**Fig. 3.**
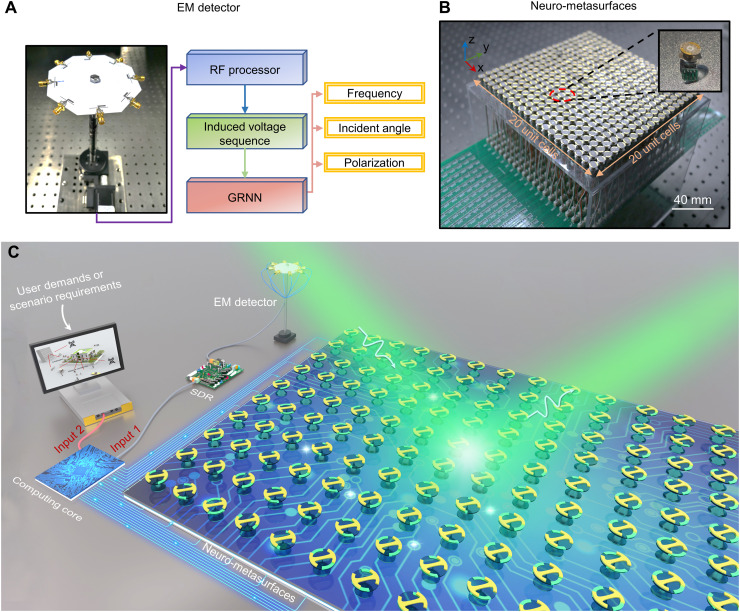
Perception-decision-action experimental setup. (**A**) Fabricated prototype of a home-made intelligent EM detector. The operating mechanism of the intelligent detector is shown on the right. An induced voltage sequence is input into the general regression neural network (GRNN) algorithm to directly obtain a complete list information of incoming wave, including frequency, incident angle, and polarization state (*47*). (**B**) Fabricated protype of the mechanical neuro-metasurfaces. Each constituent neuro-element is independently controlled by a micromotor. (**C**) A rendered photograph of the homeostatic neuro-metasurface architecture in experiment. It mainly includes an intelligent EM detector (perception), a deep learning–driven computing core (decision), and mechanical neuro-metasurfaces (action). The three parts are teamed up to automatically cater to user demands and offset surrounding dynamics. RF, radio frequency; SDR, software-defined radio.

In experiments, we build up a full set of intelligent systems that integrate the functionalities of perception, decision, and action, as shown in Fig. 3. For the perception component (Fig. 3A), we deploy an eight-port polarization and incident angle-sensitive antenna array to directly extract the full parameters of the incoming wave, assisted by a generalized regression neural network; see note S7 and Methods (*47*). For the action component (Fig. 3B), we introduce a mechanical neuro-metasurface, and each element provides an independent local reflection response via a micromotor (with a rated speed of 2000 rpm and rated power of 250 mW). Compared with conventional phased antenna, the power consumption of the neuro-metasurfaces is ~25%; see details in note S6. Compared with the lumped element tuning approach [e.g., an SMV2019-079LF commercial varactor diode has a power dissipation of 250 mW (*34*)], although their power consumptions are similar, mechanical neuro-metasurfaces execute geometric actuation only in one step without continuous energy supply (nonvolatile advantage). This way, the heat dissipation issue could be relieved to some extent, and the anti-jamming capability could be lifted in volatile environments (*48*).

**Fig. 4.**
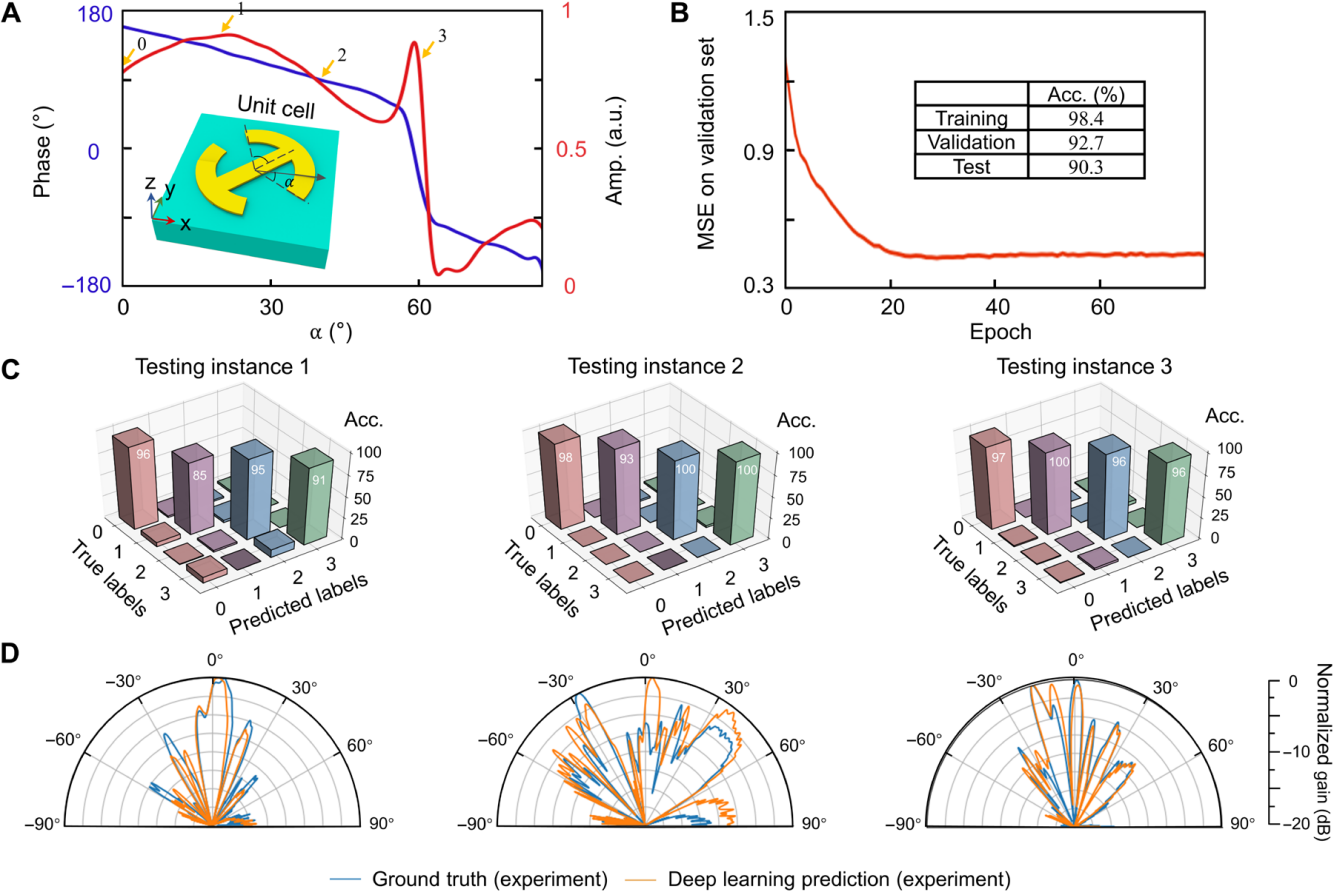
Experimental verification of homeostatic neuro-metasurfaces. (**A**) Reflected response of the neuro-metasurfaces at 13.4 GHz. The result is obtained for cross-polarized wave when the neuro-metasurfaces are illuminated by the *x*-polarized plane wave. Each element imparts a local phase (blue line) and amplitude (red line) change to the input wave as a function of the rotation angle α. The inset shows a schematic diagram of the neuro-element; see note S1 for details. a.u., arbitrary units. (**B**) Training results over the epochs. The accuracies of both the training and test sets exceed 90%, indicating that the trained network is without much overfitting. (**C**) Confusion matrices of the three randomly selected testing instances. The accuracies are 94, 97.5, and 98.25%, which is defined by the number of correctly predicted labels divided by the total number of labels. (**D**) Experimental results in the *xoz* plane for the three pairs of neuro-metasurfaces.

Without loss of generality, we design a mechanical neuro-metasurface consisting of double C-shape elements (*49*); see the schematic diagram in Fig. 4A and the geometrical parameters in fig. S1. The dimensions of the whole neuro-metasurfaces are 160 mm by 160 mm by 2 mm (20 × 20 unit cells), working within 13.1 to 13.5 GHz. For each neuro-element, two identical circular metallic patches and a central axis are etched on a 2-mm-thick F4B substrate (the relative permittivity is ε_r_ = 2.65 and the loss tangent is tanδ = 0.009). In Fig. 4A, the simulated results show that the reflected phase of the mechanical neuro-metasurfaces can be tuned in a wide range by adjusting the rotation angle of the circular metallic patch for cross-polarized wave. For the varied reflected amplitude, it only slightly affects the radiation gain, whereas the shape of the radiation remains almost identical (fig. S6). To facilitate the training of deep learning, we consider four discrete states with rotation angles of 0°,20°,40°, and 60°, corresponding to the phase shifts of 159°, 128°, 89°, and −37°. The radiation pattern with different phase quantization levels is also analyzed in note S5. We find that the radiation pattern with the four discrete states is close to that with the ideal continuous phase level. The computing time of neural network with different phase discretization level is similar (~20 ms).

### On-demand wireless channel management with homeostatic neuro-metasurfaces

By using Computer Simulation Technology (CST) Microwave Studio software and mixed-sample data augmentation (see Methods), 84,400 simulated far-field/wireless channel data at different frequencies are collected and then separated into training (80%), validation (10%), and test sets (10%). The wireless channel data are normalized and shuffled before being fed into the neural network. A classic VGG-16 architecture is identified as the base architecture to implement our encoder-decoder structure (Fig. 2B). The accuracy rates on both the training and test sets exceed 90%, indicating that the pretrained encoder-decoder is reliable without much overfitting (Fig. 4B). To test our global inverse network experimentally, we blindly choose three wireless channel characteristics from the test set (the corresponding frequencies are 13.2, 13.3, and 13.5 GHz). The selected far fields are inversely designed to generate candidate neuro-metasurface pattern with the accuracies of 94, 97.5, and 98.25%, respectively, as shown in Fig. 4C. We then measured their radar cross section (RCS), σ = 2πρ_0_∣*E_t_* − *E*_in_∣^2^/∣*E*_in_∣^2^, where *E_t_* and *E*_in_ are the observed electric field and incident electric field and ρ_0_ is set to 1.3 m for the far-field approximation. The high consistency between the RCS curves of the ground-truth and the neuro-metasurfaces in Fig. 4D lays a foundation for the following on-site experiment. The experimental efficiency (about 75%) of neuro-metasurfaces is calculated in note S11.

**Fig. 5.**
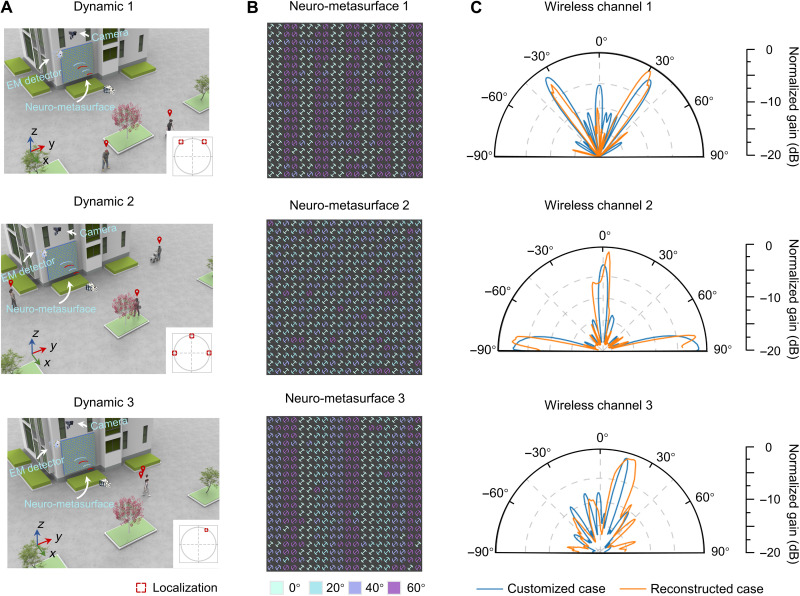
On-demand wireless channel management with homeostatic neuro-metasurfaces. (**A**) Dynamics of an on-site video at *t* = 3″29, 11″06, and 17″04; see movie S1 for the full video. The homeostatic/self-acting neuro-metasurfaces, decorated on a lateral wall, aims to enhance outdoor signals at the desired receivers or destructively at the non-intended receivers. The bottom-right inset shows the crucial signal directivities according to the locations and numbers of pedestrians. (**B**) Homeostatic neuro-metasurface reconfigurations output by the global inverse design algorithm. The homeostatic neuro-metasurfaces are characterized into four states with the rotation angles of 0^°^,20^°^,40^°^,and 60^°^. (**C**) Experimental wireless channel results (*xoy* plane) of the homeostatic neuro-metasurfaces and the customized situation.

We then progress to an on-demand wireless channel management with homeostatic neuro-metasurfaces. In addition to the incoming signal obtained by the EM detector, we also use a camera to sense the movement of pedestrians. We consider a real-world scenario near a shopping mall in the city of Hangzhou (movie S1) and randomly extract three dynamics at *t* = 3^″^29, 11^″^06, and 17^″^04, as schematically shown in Fig. 5A. In these scenarios, the homeostatic neuro-metasurfaces are assumed to be arranged on a lateral wall, with the aim of dynamically managing the wireless channel without human intervention. The wireless channel is humanized according to both the locations of pedestrians and the EM waves impinging onto the neuro-metasurface. Figure 5B shows the neuro-metasurface patterns predicted by the global inverse design algorithm, and Fig. 5C shows the measured wireless channel (*xoy* plane) together with the customized wireless channel. The high consistency in Fig. 5C strongly validates the generality of the global inverse design algorithm and the robustness of mechanical metasurfaces. Furthermore, we carry out an experiment for an off-the-shelf scenario, real-time localizing the public bus in Fig. 1 when it turns left. The homeostatic neuro-metasurfaces also exhibit accurate and agile tracking ability; see movie S2. Other complex situations are left in note S10, such as the power management for different user distances and the simultaneous changes of radiation pattern and frequency, and we show that the reflection spectra of the neuro-metasurfaces are relatively robust when the incident angle varies between −30° and 30°.

## DISCUSSION

In conclusion, we have synergized mechanical-actuating metasurfaces with deep learning to usher in an era of EM smart cities and infrastructures and for indoor applications with a high density of users, such as in stadiums and airports (*6*). No human intervention and iterative computation are involved in the experiment. On a fundamental level, we bestow the neuro-metasurfaces with the generic ability to independently analyze and solve problems, rather than fixed functionalities for fixed environments and incoming waves. This self-learning ability is of paramount importance for numerous applications, such as invisibility cloaks and biological imaging in random media (*50*). A similar concept can be readily extended to higher frequencies with the proposed global inverse metasurface design and mature micro/nano fabrication technology, such as microelectromechanical systems technique and phase-change materials. We believe that the global inverse design strategy is a unique advantage, which, in combination with optical active metasurfaces, may be key to making various intelligent metadevices (*51*).

Looking forward, it would be nothing short of astonishing to migrate scenarios, e.g., from smart cities to smart offices, by sharing common experiences and parameters in transfer learning (*52*). Another meaningful improvement would be the use of semisupervised and unsupervised learning, which would largely relax the high reliance on massive data collection for even larger-scale neuro-metasurfaces. For a more general open-loop operation system, the on-site learning working mode can be applied to regulate wireless channel, providing robustness to unexpected stimuli (*53*–*54*). In turn, we also anticipate that the homeostatic neuro-metasurfaces will accelerate deep learning algorithm in optics by harnessing the advantages of parallel computing and speed-of-light operation (*15*).

## METHODS

### Data generation

The training data are obtained with the commercial software CST Microwave Studio. For the numerical simulation, the actual structures for the designed metasurfaces are adopted. A total of 84,400 sets of metasurfaces are generated in the multiparadigm numerical computing tool MATLAB and then transferred into the commercial software package CST Microwave Studio for continuous automatic full-wave simulations via the MATLAB-CST cosimulation method.

### Augmentation technique

The dataset is expanded by the mixed-sample data augmentation method and random variation data augmentation method. The core idea of the mixed-sample data augmentation method is to randomly mix two training labels (metasurface arrangements) at a certain rate to generate new data, while the random variation data augmentation method involves changing the training labels randomly at a certain variation rate. Starting with 25 representative training labels, the dataset is expanded to 84,400 items by using the method described above with a mixing rate and variation rate that vary from 0 to 100%, thus effectively increasing the diversity of the samples and improving the robustness of the model. During the training period, Gaussian noise and random rotation are randomly applied to enhance the generalization capability of the model.

### Intelligent EM detector

A homemade intelligent EM detector is composed of an eight-port antenna array for simultaneously attaining frequency, directional-of-arrival, and polarization data. The eight-port metasurface antenna array is connected to two radio frequency switches (HMC641ALC4) and used to collect the amplitude-only sequence from ports 1 to 8 on a microsecond time scale. The received signal is amplified by a broadband amplifier and down-converted to 0.2 to 4 GHz. Then, we use an AD9361 as the radio frequency processor, which contains a low-noise amplifier, mixer, and other electric components, and use a Xilinx Zynq for data processing with a calculation accelerator assisted by Field Programmable Gate Array (FPGA). On the basis of the collected data, a generalized regression neural network outputs the frequency, directional of arrival, and polarization of the received signal; see note S7. The entire detection takes about 60 ms, including 35 ms for frequency sweeping, 10 ms for the machine learning calculation, and 15 ms consumed by other data-processing algorithms, such as fast Fourier transform and median filter.

### Experimental measurement

The experiment is carried out in an anechoic chamber, which mainly includes a transmitting horn antenna, a receiving horn antenna, and an intelligent EM detector. In far-field measurements, both the transmitting and receiving horn antennas are fixed on an arch-shaped bracket with a radius of 1.3 m and digitally controlled to rotate within 0-π. The receiving horn antenna is connected to a vector network analyzer to detect the scattered field, including the amplitude and phase information. The far-field experimental setup is shown in note S8.

### Running time

The total consuming time of the neuro-metasurfaces includes three parts: detection time (~60 ms), calculation time (~20 ms), and execution time (~5 ms), when the complexity of the input/environment complexity increase does not affect the action time greatly. For example, when the dimension of input channel increases from 200 × 200 to 400 × 400, the action time only increases from ~20 to ~25 ms (with an additional convolutional layer).

## Supplementary Materials

### This PDF file includes:

Notes S1 to S11 Figs. S1 to S13 References
