# Supplementary material for: Homeostatic neuro-metasurfaces for dynamic wireless channel management
Source: Sci Adv. 2022 Jul 6;8(27):eabn7905. doi: 10.1126/sciadv.abn7905 (PMC9258947; doi:10.1126/sciadv.abn7905)
Supplement: Supplementary file 1 — Notes S1 to S11 Figs. S1 to S13 References [file sciadv.abn7905_sm.pdf]

Science Advances
AAAS

Supplementary Materials for

Homeostatic neuro-metasurfaces for dynamic wireless channel management

Zhixiang Fan *et al.*

Corresponding author: Chao Qian, chaoq@intl.zju.edu.cn; Bin Zheng, zhengbin@zju.edu.cn; Hongsheng Chen, hansomchen@zju.edu.cn

Sci. Adv. **8**, eabn7905 (2022)
DOI: 10.1126/sciadv.abn7905

The PDF file includes:

Notes S1 to S11
Figs. S1 to S13
Legends for movie S1 and S2
References

Other Supplementary Material for this manuscript includes the following:

Movies S1 and S2

Supplementary Note 1: Design and reflection spectra of the neuro-metasurfaces

The basic unit cell (meta-atom) of the neuro-metasurfaces is illustrated in Fig. S1. Two identical circular metallic patches and a C-shaped metallic particle are etched on a 2 mm-thick F4B substrate (with the relative permittivity of $\varepsilon_r = 2.65$, and loss tangent of $\tan\delta = 0.009$). The metallic layer is modeled as a copper film with a thickness of 0.018 mm (the electrical conductivity is $\sigma = 5.96 \times 10^7 \mathrm{S/m}$). We can adjust the reflection spectra through varying the rotation angles of the two circular metallic patches.

Figure S2 plots the reflected phase and amplitude under four different rotation angles (working under polarization conversion case). At the frequency band of 13.1–13.5 GHz, the reflected phase almost covers 0-2π, while the reflected amplitude remains high.

In our work, the physical dimension of the neuro-metasurfaces is set to $160 \times 160 \times 2\ \mathrm{mm}^3$, composed of 20×20 meta-atoms. For on-demand wireless channel management, we utilized a micro-motor to control the rotation angle of the metallic patch (homeostatic neuro-metasurfaces). Ultimately, 20×20 motor array is designed to control each meta-atom independently. Supplementary Note 8 provides a detailed experimental description.

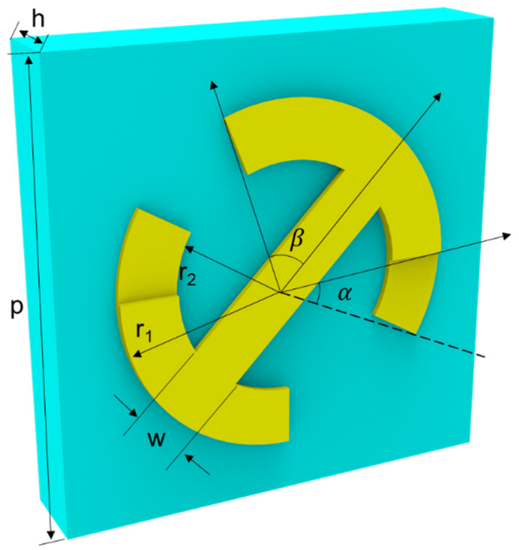

Figure S1 | Three-dimensional (3D) illustration of the unit cell. The detailed parameters are as follows: P = 8 mm, h = 2 mm, w = 1 mm, r_1 = 3 mm, r_2 = 2 mm and $\beta = 62.5^\circ$. The reflection spectrum can be tuned via the rotation angle α.

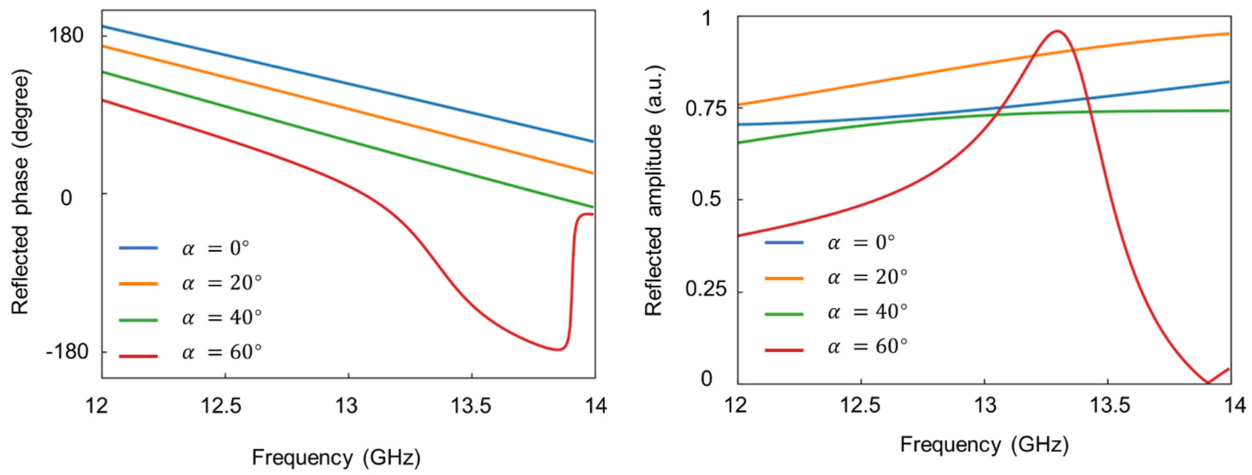

Figure S2 | Reflection spectrum of the unit cell. Reflected phase and amplitude of the cross-polarized wave under normal x-polarized wave incidence. Colored lines represent different orientation angles. The pale yellow area corresponds to the working frequency band 13.1–13.5 GHz.

Supplementary Note 2: Data preprocessing of the wireless channel

Far-field analysis can provide valuable yet mainly qualitative information about the impact of errors. To quantify performance degradation and extract behavioral trends, a set of performance metrics is used. In general, the following performance metrics are considered important far-field metrics, as defined in [55]:

- Half Power Beam Width (HPBW): In a plane containing the maximum direction of a beam, the HPBW is the angle between the two directions in which the radiation intensity is one-half the value of the beam. It is a widely-used factor to describe beam width.
- Directivity (D): The ratio of the radiation intensity in a given direction from the antenna to the radiation intensity averaged over all directions. The average radiation intensity is equal to the total power radiated by the antenna divided by 4π.
- Radiation Pattern Lobes (RPLs): The various parts of a radiation pattern are referred to as lobes, which may be subclassified into major/main, minor, side and back lobes. A radiation lobe is a portion of the radiation pattern bounded by regions of relatively weak radiation intensity. The major lobe is defined as the radiation lobe containing the direction of maximum radiation. A minor lobe is any lobe other than the major lobe. A side lobe is a radiation lobe in any direction other than that of the intended lobe. A back lobe is a radiation lobe whose axis makes an angle of approximately $180°$ with respect to the beam of an antenna.

- Side Lobe Levels (SLLs): Minor lobes usually represent radiation in undesired directions, and they should be minimized. The level of a minor lobe is usually expressed as the ratio of the power density in the lobe in question to that of the major lobe. This ratio is often termed the "side lobe level".

In this Article, the objective of the global inverse design model is to extract characteristics from the wireless channel and finally output the corresponding neuro-metasurface distribution. Therefore, it is of great importance to conduct feature engineering in advance. Figure S3 shows the data preprocessing method utilized for the far-field pattern in a one-dimensional (1D) example. RCS_{upmask} and $RCS_{submask}$ are two arrays representing the 3 dB down boundary and 15 dB down boundary of RCS, respectively. From Fig. S3, we can see that RCS_{upmask} shows the HPBW of the radiation pattern, while $RCS_{submask}$ plays an important role in the RPLs and SLLs. With all three arrays, RCS, $RCS_{submask}$, and RCS_{upmask}, the above performance metrics, including the beam direction, directivity, radiation pattern lobes, and side lobe levels, can be easily obtained [56].

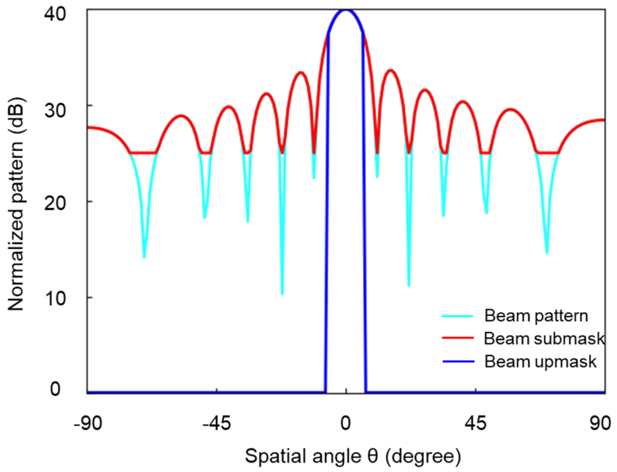

Figure S3 | One-dimensional (1D) example of the radiation, submask, and upmask patterns. The light blue line, red line and deep blue line represent the beam pattern, beam submask pattern and beam upmask pattern, respectively. The following performance metrics can be obtained easily through the beam mask preprocessing: the beam direction, directivity, radiation pattern lobes, and side lobe levels.

Supplementary Note 3: Architecture of the global inverse design model

As discussed in the main text, the output of the encoder-decoder network is 20 × 20 metasurfaces with a labeled state 0/1/2/3. The input of the encoder-decoder network is composed of two parts:

radiation pattern and incident information. We decomposed the radiation pattern (expressed in polar coordinates) into three feature extraction pipes (pattern, upmask, and submask pipes), each of which has a dimension of 200 × 200. The incident information includes the frequency, incident angle, and polarization, each of which also has a dimension of 200 × 200. Figure S4 shows a specific input example in the experiment, where the incident angle and polarization are not involved.

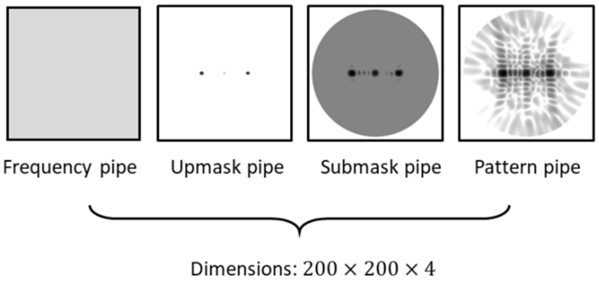

Figure S4 | One specific example of the input.

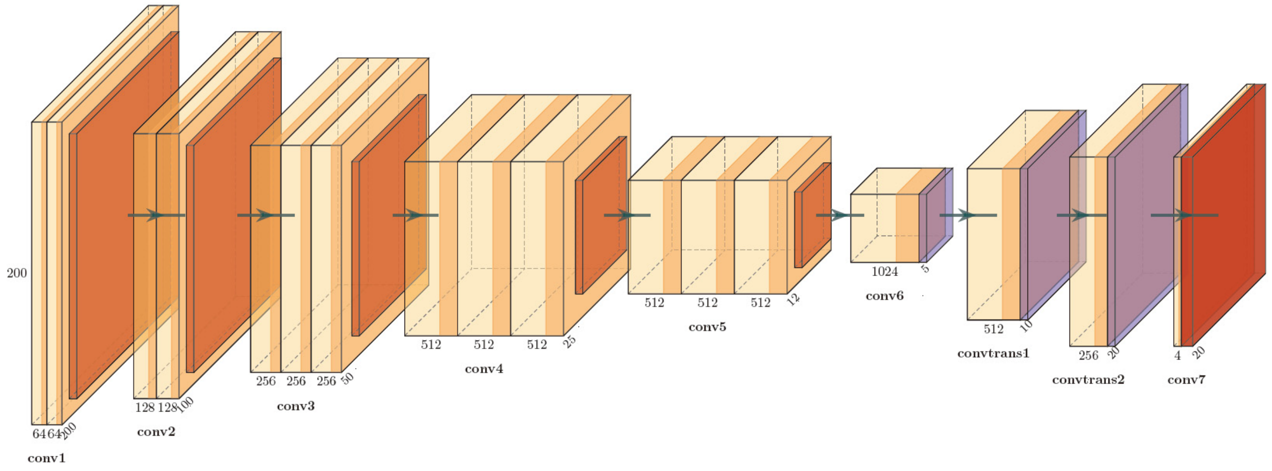

Figure S5 | Architecture of the applied global inverse design model. The global inverse design model consists of 15 convolutional layers, 2 transposed convolutional layers, 5 pooling layers and 3 batch normalization layers.

To build up a mapping from radiation pattern to neuro-metasurface arrangement, we design a fully convolutional network (FCN) [57], which has been widely used in the semantic segmentation field. As shown in Fig. S5, the FCN consists of 15 convolutional layers, 2 transposed convolutional layers, 5 pooling layers and 3 batch normalization layers. The network can be divided into two parts: an encoder part and a decoder part. In the first part, the radiation pattern matrix undergoes dimensionality reduction from $200 \times 200 \times 4$ to $5 \times 5 \times 1024$; this part is used to extract the characteristics of radiation pattern data. In the second part, the matrix has its dimensions increased

from $5 \times 5 \times 1024$ to $20 \times 20 \times 4$, and it is used to adjust the extracted feature matrix to conform with the size of the output. The encoder part is constructed by the VGG-16 network, while the decoder part is constructed by transposed convolutional blocks, including transposed convolutional layers and batch normalization layers.

Supplementary Note 4: Training process of the global inverse design model

The 84,400 collected samples are divided into training, validation and test sets (80%, 10% and 10%, respectively). Among them, the training data are used to generate gradients and optimize the weights, the validation data are used as a stopping condition in case of overfitting, and the test data are used as a final marker. The input variables include the preprocessed far-field pattern and frequency information and the corresponding output rotation angles of all elements. The input data are normalized before being fed into the input layer because this step is helpful for convergence.

When training the FCN, the weights are initialized via Glorot initialization with a uniform distribution $W \sim U(-\sqrt{\frac{6}{fan_{in}+fan_{out}}}, \sqrt{\frac{6}{fan_{in}+fan_{out}}})$ for all layers [58], where fan_{in} and fan_{out} are the numbers of inputs and outputs, respectively. A ReLU activation function and the Adam optimization algorithm are used during training [59]. We terminate the training process when the validation error stops decreasing over 80 epochs with a learning rate of 0.0001, and finally, the architecture with the lowest validation error is selected. The above training procedure is implemented using Python version 3.7.6 and the TensorFlow framework (version 2.1.0) on a server (Tesla P100-PCIE-16 GB GPU running on a Linux operating system).

To evaluate the effectiveness of the global inverse design model, two evaluation indices are used, including the cross-entropy loss and accuracy. The cross-entropy loss is defined as:

$$L_{log}(Y,P) = -\frac{1}{MN}\sum_{i=0}^{M-1}\sum_{j=0}^{N-1}\sum_{k=0}^{K-1} y_{i,j,k} \log p_{i,j,k} \quad \text{(Eq. S1)}$$

where *M*, *N*, and *K* are the numbers of meta-atoms in the metasurface, training data and types of rotation angles, respectively, while $p_{i,j,k}$ is the probability that the rotation angle of the *i*-th element of the *j*-th sample is the *k*-th rotation angle. The other evaluation index is accuracy, defined as:

$$Acc = \frac{1}{N}\sum_{j=0}^{N-1} Acc_j \quad \text{(Eq. S2)}$$

where N is the number of training data and Acc_j is the accuracy of the *j*-th sample.

Supplementary Note 5: Performance influenced by amplitude variation and phase discretization

In this note, we discuss the output performance influenced by amplitude variation and phase discretization. For the amplitude variation, the reflection amplitude of neuro-metasurface is not very close to unity (Fig. 4a of the main text). Such amplitude variation only affects the radiation gain, whereas the shape of the radiation remains almost identical. To better illustrate this point, we compared two metasurfaces with unitary amplitude and varied amplitude. Figure S6 showcases the radiation pattern for two groups of metasurfaces. The quantitative calculations indicate that the radiation gain of the metasurfaces with varied amplitude reaches 86.8% (Fig. S6a) and 86.1% (Fig. S6b), compared with that with unitary amplitude.

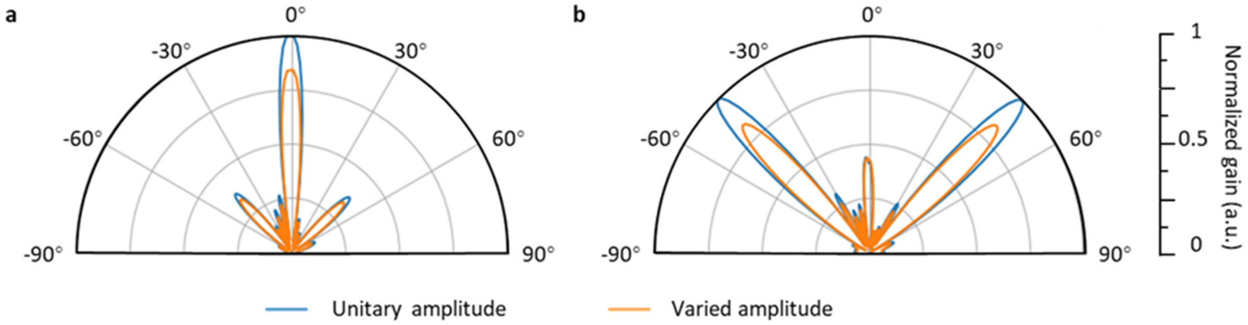

Figure S6 | Comparisons of the metasurfaces with unitary amplitude and varied amplitude.

For the phase discretization, we discuss the influence of quality and time. First, we analyzed the quality of radiation pattern influenced by the phase discretization. As listed in Fig. S7a, we considered four situations: 2 phase quantization level (0 and 6), 4 phase quantization level (0, 2, 4, and 6), 8 phase quantization level (0-7), and continuous phase variation. Note that the data in Fig. S7a are extracted from the simulation results, including both amplitude and phase. Figure S7b depicts the radiation pattern, where the blue, orange, green, and red lines represent the results by 2/4/8 phase quantization levels and continuous phase variation. All four situations aim to reconstruct the target beam with 34° deflection. The results indicate that, when the phase quantization levels reach 4 phase quantization levels, the beam quality is close to the continuous phase level.

Regarding the influence on time, the neural network for different phase quantization levels can be constructed using a similar network structure, with only a slight difference in the last few convolutional layers, which does not significantly affect the computing time. To validate this, we built neural networks of 2/4/8 phase quantization levels (with 2/4/8 channel of last convolution layers) for

inspection. The forward prediction time is ~20 ms, in line with our expectations. For the case of continuous phase variation, we also built a neural network for inspection, with the forward prediction time of ~30 ms. Thus, we conclude that phase discretization does not have a significant impact on computing time.

a

State	Rotation angle	Amplitude	Phase
0	0°	0.73	159°
1	10°	0.86	144°
2	20°	0.89	128°
3	30°	0.85	108°
4	40°	0.74	89°
5	50°	0.6	70°
6	60°	0.71	−37°
7	80°	0.22	−141°

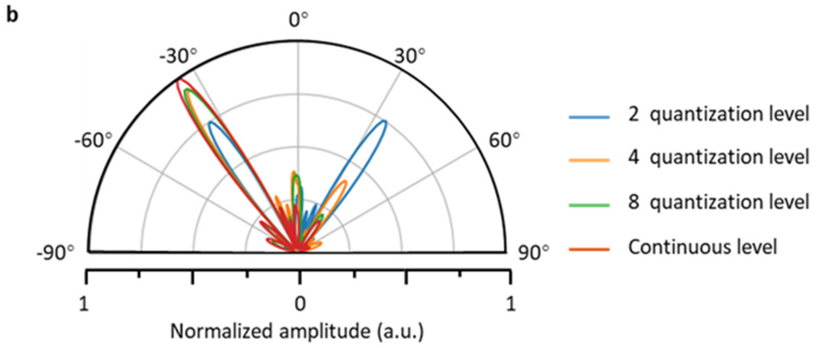

Figure S7 | Comparison of radiation patterns of metasurfaces with different phase quantization levels and continuous phase variation. a, Reflection characteristics of the discrete selected meta-atom at 13.2 GHz. **b**, Radiation pattern with different phase quantization levels.

Supplementary Note 6: Energy consumption of the neuro-metasurfaces

We calculated the energy consumption of the neuro-metasurfaces and compared it with conventional phased antenna array. The power consumption of the neuro-metasurfaces is ~25% that of conventional phased antenna array. More importantly, owning to the non-volatile advantage, the practical energy consumption is smaller than 25%. The following is the detailed analysis.

A typical phased array antenna architecture is shown in Fig. S8a, and the power consumption of individual electronic components is listed in Fig. S8b [60-61]. The total power consumption of the phased array antenna P_{PAA} is given as follows:

$$P_{PAA} = P_{ADC} + P_M + P_{VGA} + N \times (P_{PS} + P_{PA}) \approx N \times P_{PA} \quad \text{(Eq. S3)}$$

where N represents the number of the antenna elements. From Fig. S8b, we can see that P_{PAA} can be mainly determined by the number of the power amplifiers ($P_{PA} \approx 1\ W$).

For the neuro-metasurfaces, the total power consumption P_{HN} mainly comes from the micro motors and the detector (e.g., LNA and mixer), which can be calculated as follows:

$$P_{HN} = P_{LNA} + P_M + N \times P_{MM} \approx N \times P_{MM} \quad \text{(Eq. S4)}$$

where N represents the number of the metasurface elements (same as the number of the antenna elements). Thus, P_{HN} is mainly determined by the number of the micro motors ($P_{MM} \approx 0.25\ W$), which is only $\sim$25% that of the phased array antenna.

Moreover, we would like to emphasize another point that the neuro-metasurfaces can work without a continuous energy supply (non-volatile). This indicates that, in a steady state and static scenario, the practical power consumption will be much smaller than $0.25\ W$ (more energy-efficient).

a

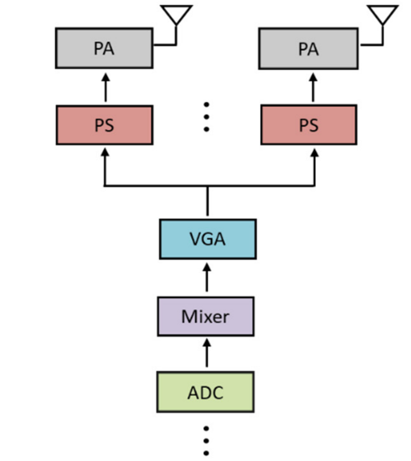

b

Label	Device	Power consumption
P_{PS}	Phase shifter (PS)	$2\ mW$
P_{PA}	Power amplifier(PA)	$1\ W$
P_{VGA}	Variable gain amplifier(VGA)	$2\ mW$
P_M	Mixer	$0.3\ mW$
P_{ADC}	Analog to digital converters (ADC)	$15\mu W/\text{GHz} \cdot f_s 2^{ENOB}$
P_{LNA}	Low-noise amplifier (LNA)	$5.4\ mW$
P_{MM}	Micro motor (MM)	$250\ mW$

Figure S8 | Comparison of power consumption between conventional phased array antenna and neuro-metasurfaces. a, Architecture of conventional phased antenna. It mainly includes PS, PA, VGA, mixer, and ADC (power consumption of the ADC is dependent on the effective number of bits (ENOB) and the sampling frequency f_s). **b**, Power consumption of individual electronic devices**.**

Supplementary Note 7: The GRNN in the intelligent EM detector

The intelligent EM detector is physically made of a hexadecagonal eight-port antenna array, which can perceive the incident information related to frequency, polarization and incident angle in real time. The frequency component can be retrieved via the Fourier transform of the time-varying signal. For the polarization and incident angle components, we deployed a generalized regression neural network (GRNN) for inverse reasoning, with a set of labeled data $\{\boldsymbol{a}(i), \boldsymbol{s}(i) | i = 1, \dots, N\}$ (N is the number of the labeled data). As shown in Fig. S9, the GRNN contains an input layer, pattern layer, summation layer, and output layer. Consider an input vector $\boldsymbol{a}(u)$ is fed to the pattern layer, each summation layer node L_b(except for the red node L_r; the red node in the summation layer is the

sum of pattern layer outputs) calculates the weighted sum of the pattern layer outputs using labeled data γ_{ij}. The output of the pattern layer (red node in the summation layer) is given as follows:

$$L_r = \sum_{j=1}^{N} e^{-\frac{\|a(u)-a(j)\|}{2\delta^2}} \quad \text{(Eq. S5)}$$

where δ is a hyperparameter of the GRNN controlling the influence of the basis functions. L_r is the red node in the summation layer, and other green nodes read,

$$L_g = \sum_{j=1}^{N} \gamma_{ij} e^{-\frac{\|a(u)-a(j)\|}{2\delta^2}} \quad \text{(Eq. S6)}$$

where $i = 1, \ldots, N$. The prediction result is $s = L_g/L_r$. When a suitable number of labeled data are collected, the signal captured by the detector can be processed by the GRNN to predict the information of incidence and polarization incoming wave with high fidelity.

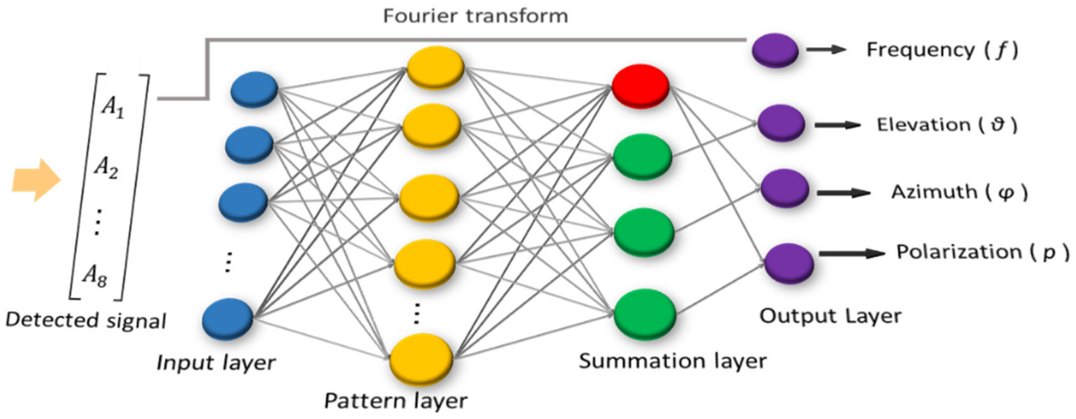

Figure S9 | Schematic of the GRNN used in the intelligent EM detector. The GRNN contains an input layer, pattern layer, summation layer, and output layer.

Supplementary Note 8: Experimental measurement

The experiment was carried out in an anechoic chamber. As shown in Fig. S10, it mainly includes a homeostatic neuro-metasurface, an intelligent EM detector, and a control circuit. The homemade intelligent EM detector was composed of an 8-port metasurface array for simultaneous attainment of the frequency, incident angle, and polarization. According to the information of incoming waves and on-demand wireless channel, the control circuit independently controls each micro-motor (in total, 20×20 micro-motors). In the measurement, a receiver antenna moves along an arch (the radial distance between the neuro-metasurfaces and the receiver antenna is fixed at 1.3m) to obtain the RCS (radar cross-section). The experimental results in Figs. 4 and 5 are obtained in this way.

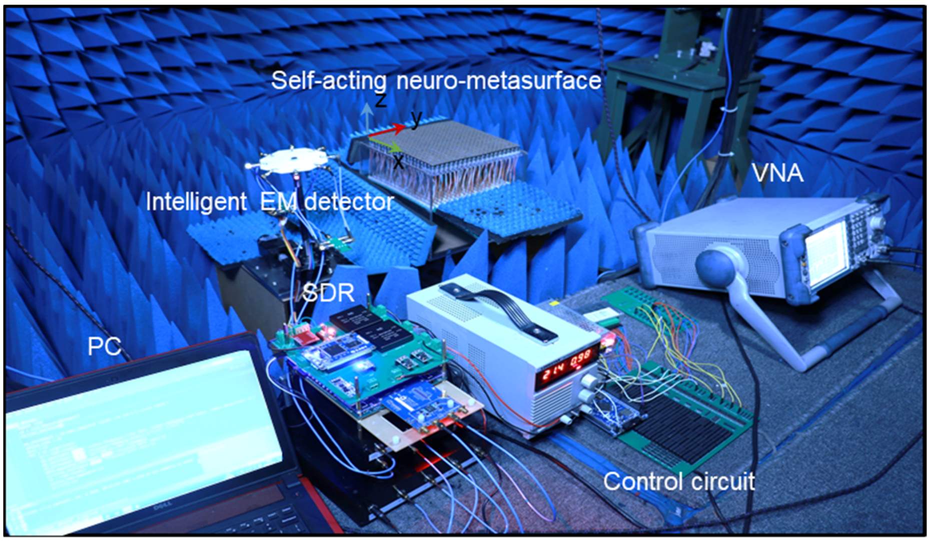

Figure S10 | Experimental setup. VNA, vector network analyzer. SDR, software-defined radio. PC, personal computer.

Supplementary Note 9: Bandwidth management of the neuro-metasurfaces

Two bandwidths are involved in wireless communication, i.e., the bandwidth of the user channel and the working bandwidth of the metasurface. Below, we will discuss them one-by-one.

The bandwidth of the user channel can be certainly managed, depending on the communication parties via different communication protocols. It is not directly related to the metasurfaces, because metasurfaces here are used to manipulate the received signal rather than actively transmitting the signal. Thus, we only need to guarantee that the working bandwidth of the metasurfaces is larger than the bandwidth of the user channel.

The working bandwidth of the metasurface is physically determined by the metasurface structure; for example, our double C-shaped metasurfaces work within 13.1–13.5 GHz. For a wider bandwidth, we need to design other metasurfaces for specific requirements. However, we would like to emphasize that the 0.4 GHz bandwidth here is considerably large for a high communication rate; for example, accommodating 20 standard IEEE 802.11n WiFi channels (20 MHz for each user channel). Furthermore, we can improve the data allocation efficiency through methods of frequency division multiplexing, time division multiplexing, and space division multiplexing. For example, by designing space-time-coding matrixes, the metasurface can independently manipulate the propagation direction of multiple harmonic waves to implement both space- and frequency-division multiplexing [12]. In addition, we may consider the communication network constructed with multiple

metasurfaces (with different bandwidth) to jointly increase the flexibility of the on-demand resource allocation.

Supplementary Note 10: More application scenarios with the neuro-metasurfaces

In the design process, we also included power management in the radiation pattern decision. It means that for different user distances, the neuro-metasurfaces can assign different power. To better illustrate this point, we carried out another experiment in Fig. S11. Despite the similar shapes of the three radiation patterns, the assigned powers were different (1:0.75:0.5 for different user distances). The result clearly shows that neuro-metasurfaces can regulate different power assignments for different user distances.

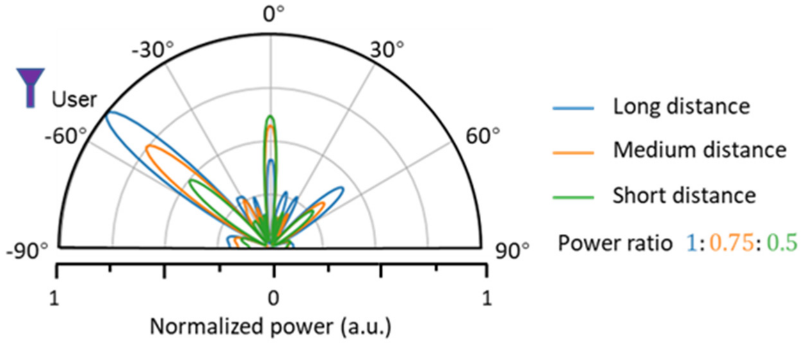

Figure S11 | On-demand power management for varying distances. The user distances were set as long, medium, and short distances, with assigned powers of 1, 0.75, and 0.5, respectively, and an azimuth angle of -50°.

In the experiment of Fig. 5, the detected frequency, polarization, and incident angle of EM wave are unchanged. Having said that, it does not mean that the neuro-metasurfaces can only work under a fixed EM incidence. We demonstrated a scenario in which one or more of the parameters are varied, e.g., the incident frequency and radiation pattern are simultaneously varied. Figure S12 clearly shows that the reconstructed radiation patterns are in consistent with the target ones, even for different frequencies. Regarding the changes in the incident angle, we simulated the reflection spectra of the neuro-metasurfaces in different incident angle θ. As shown in Fig. S13, the reflection phase of the metasurfaces remains almost unchanged when the incident angle varies between -30° ~ 30°. Therefore, the neuro-metasurfaces is robust for wide incident angle in real-scenario. As for the change of polarization, they cannot be freely manipulated due to the inherent constraint of double C-shaped metasurfaces that work for cross-polarization. It's expected that by introducing different

metasurfaces structure, we can achieve neuro-metasurfaces for different EM manipulation, such as Pancharatnam-Berry metasurfaces for LCP/RCP incidence [62, 63]. This will be another important work in this field but will not alter the qualitative behavior of the neuro-metasurfaces demonstrated here.

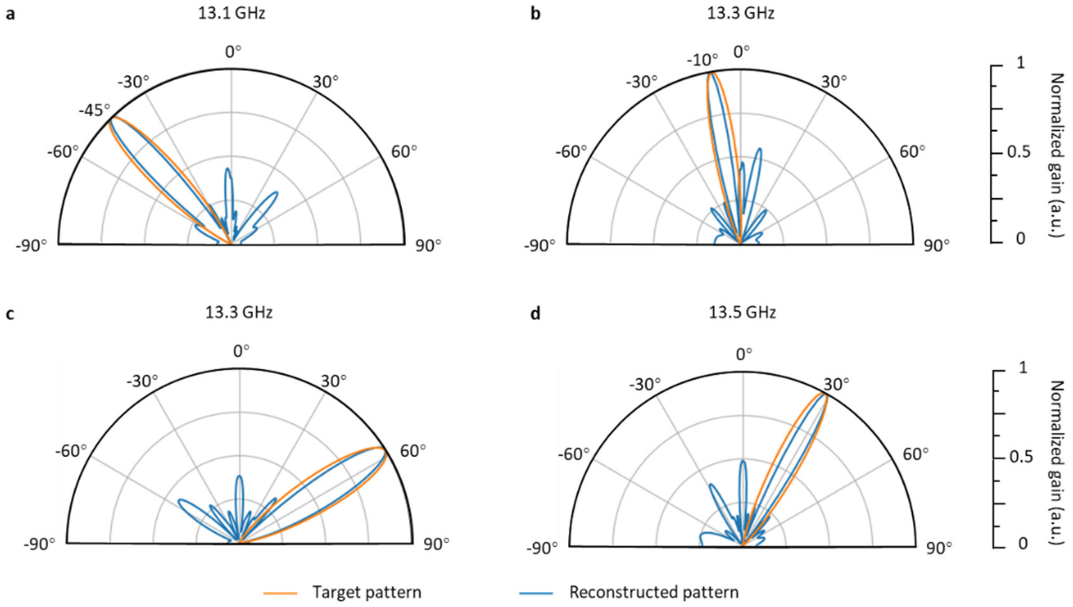

Figure S12 | On-demand wireless channel management with varying frequency and radiation pattern.

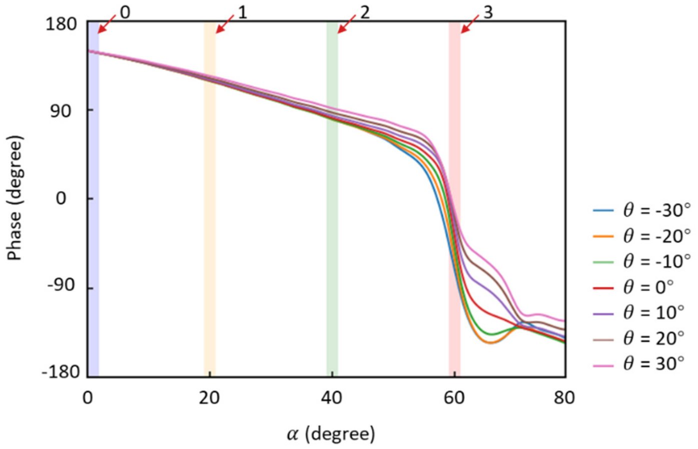

Figure S13 | Reflected response of the neuro-metasurfaces under different incident angle θ.

Supplementary Note 11: Working efficiency of the neuro-metasurfaces

We calculated the experimental/simulation efficiency as the ratio between the power carried by the desired wave and the power of the incident wave [64]The efficiency is mathematically expressed as $\eta = \frac{P_{ref}}{P_{tot}} \times \frac{P_{des}}{P_{ref}}$, where $\frac{P_{ref}}{P_{tot}}$ represents the ratio between the power carried by the total reflected

wave and that of the incident wave, $\frac{P_{des}}{P_{ref}}$ represents the ratio between the power carried by the desired reflected wave and that of the total reflected wave. $\frac{P_{des}}{P_{ref}}$ is calculated by $\frac{P_{des}}{P_{ref}} = \frac{\oint \hat{n} \cdot \vec{S}\, ds_1}{\oint \hat{n} \cdot \vec{S}\, ds_2}$, where $\hat{n} \cdot \vec{S}$ can be approximately evaluated by $\left|\vec{E}\right|^2$ [37]. It is very difficult to perform three-dimensional integration over the reflected waves in the experiment. Therefore, we chose two-dimensional typical planes to perform the integration over the reflected waves. Taking the predicted results in Fig. 4d of the main text as examples, the three experimental efficiencies are 77.9%, 70.9% and 72.3%, corresponding to the simulation efficiencies of 80.7%, 76.8% and 79.1%, respectively.

Other supplementary information for this manuscript includes:

- Supplementary Video S1 (.mp4 format). To check the generality of our neuro-metasurfaces, we recorded an on-site video near a mall in the city of Hangzhou. Specifically, we extracted three frames at $t = 3''29$, $11''06$, and $17''04$ as the research objects of the neuro-metasurfaces.
- Supplementary Video S2 (.mp4 format). We take Fig. 1 in the main text as a demonstration, and activated the scenario. In the dynamics, a public bus turns left at the crossroads. This video vividly shows how the neuro-metasurfaces localize the public bus in real time.

REFERENCES AND NOTES

1. A. Zanella, N. Bui, A. Castellani, L. Vangelista, M. Zorzi Internet of things for smart cities. *IEEE Internet Things J.* **1**, 22–32 (2014).

2. C. Kaspar, B. J. Ravoo, W. G. van der Wiel, S. V. Wegner, W. H. P. Pernice The rise of intelligent matter. *Nature* **594**, 345–355 (2021).

3. S. Mohanty, U. Choppali, E. Kougianos, Everything you wanted to know about smart cities: The Internet of things is the backbone. *IEEE Consum. Electron. Mag.* **5**, 60–70 (2016).

4. E. Basar, Reconfigurable intelligent surface-based index modulation: A new beyond MIMO paradigm for 6G. *IEEE Trans. Wirel. Commun.* **68**, 3187–3196 (2020).

5. H. Yang, Z. Xiong, J. Zhao, D. Niyato, L. Xiao, Q. Wu, Deep reinforcement learning-based intelligent reflecting surface for secure wireless communications. *IEEE Trans Wirel Commun.* **20**, 375–388 (2021).

6. R. Napolitano, W. Reinhart, J. P. Gevaudan Smart cities built with smart materials. *Science* **371**, 1200–1201 (2021).

7. P. Del Hougne, M. Fink, G. Lerosey, Optimally diverse communication channels in disordered environments with tuned randomness. *Nat. Electron.* **2**, 36–41 (2019).

8. Q. Ma, G. D. Bai, H. B. Jing, C. Yang, L. Li, T. J. Cui Smart metasurface with self-adaptively reprogrammable functions. *Light Sci. Appl.* **8**, 98 (2019).

9. O. Tsilipakos, A. C. Tasolamprou, A. Pitilakis, F. Liu, X. Wang, M. S. Mirmoosa, D. C. Tzarouchis, S. Abadal, H. Taghvaee, C. Liaskos, A. Tsioliaridou, J. Georgiou, A. Cabellos-Aparicio, E. Alarcón, S. Ioannidis, A. Pitsillides, I. F. Akyildiz, N. V. Kantartzis, E. N. Economou, C. M. Soukoulis, M. Kafesaki, S. Tretyakov Toward intelligent metasurfaces: The progress from globally tunable metasurfaces to software-defined metasurfaces with an embedded network of controllers. *Adv. Opt. Mater*. **8**, 2000783 (2020).

10. N. Yu, F. Capasso, Flat optics with designer metasurfaces. *Nat. Mater.* **13**, 139–150 (2014).

11. T. Cai, G. M. Wang, S. W. Tang, H. X. Xu, J. W. Duan, H. J. Guo, F. X. Guan, S. L. Sun, Q. He, L. Zhou High-efficiency and full-space manipulation of electromagnetic wave fronts with metasurfaces. *Phys. Rev. Appl.* **8**, 034033 (2017).

12. L. Zhang, M. Z. Chen, W. Tang, J. Y. Dai, L. Miao, X. Y. Zhou, S. Jin, Q. Cheng, T. J. Cui A wireless communication scheme based on space- and frequency-division multiplexing using digital metasurfaces. *Nat. Electron.* **4**, 218–227 (2021).

13. Q. Wu, R. Zhang, Towards smart and reconfigurable environment: Intelligent reflecting surface aided wireless network. *IEEE Commun Mag.* **58**, 106–112 (2020).

14. H. Ren, X. Fang, J. Jang, J. Bürger, J. Rho, S. A. Maier Complex-amplitude metasurface-based orbital angular momentum holography in momentum space. *Nat. Nanotechnol.* **15**, 948–955

(2020).

15. C. Qian, X. Lin, X. Lin, J. Xu, Y. Sun, E. Li, B. Zhang, H. Chen Performing optical logic operations by a diffractive neural network. *Light Sci. Appl.* **9**, 59 (2020).

16. G. Qu, W. Yang, Q. Song, Y. Liu, C. W. Qiu, J. Han, D. P. Tsai, S. Xiao Reprogrammable meta-hologram for optical encryption. *Nat. Commun.* **11**, 5484 (2020).

17. N. M. Estakhri, B. Edwards, N. Engheta, Inverse-designed metastructures that solve equations. *Science* **363**, 1333–1338 (2019).

18. J. Jiang, M. Chen, J. A. Fan, Deep neural networks for the evaluation and design of photonic devices. *Nat. Rev. Mater*. **6**, 679–700 (2021).

19. Z. Liu, D. Zhu, K. T. Lee, A. S. Kim, L. Raju, W. Cai Compounding meta-atoms into metamolecules with hybrid artificial intelligence techniques. *Adv. Mater.* **32**, 1904790 (2020).

20. W. Ma, Y. Liu, A data-efficient self-supervised deep learning model for design and characterization of nanophotonic structures. *Sci. China Phys. Mech.* **63**, 284212 (2020).

21. H. Ren, W. Shao, Y. Li, F. Salim, M. Gu, Three-dimensional vectorial holography based on machine learning inverse design. *Sci. Adv.* **6**, eaaz4261 (2020).

22. P. R. Wiecha, O. L. Muskens, Deep learning meets nanophotonics: A generalized accurate predictor for near fields and far fields of arbitrary 3D nanostructures. *Nano Lett.* **20**, 329–338 (2020).

23. W. Ma, F. Cheng, Y. Xu, Q. Wen, Y. Liu Probabilistic representation and inverse design of metamaterials based on a deep generative model with semi-supervised learning strategy. *Adv. Mater.* **31**, 1901111 (2019).

24. W. Ma, F. Cheng, Y. Liu Deep-learning enabled on-demand design of chiral metamaterials. *ACS Nano* **12**, 6326–6334 (2018).

25. W. Ma, Y. Xu, B. Xiong, L. Deng, R. W. Peng, M. Wang, Y. Liu Pushing the limits of functionality—Multiplexing capability in metasurface design based on statistical machine learning. *Adv. Mater*. **34**, 2110022 (2022).

26. L. Raju, K. T. Lee, Z. Liu, D. Zhu, M. Zhu, E. Poutrina, A. Urbas, W. Cai Maximized frequency doubling through the inverse design of nonlinear metamaterials. *ACS Nano* **16**, 3926–3933 (2022).

27. X. Chen, Z. Wei, M. Li, P. Rocca A review of deep learning approaches for inverse scattering problems (Invited review). *Prog. Electromagn. Res.* **167**, 67–81 (2020).

28. Z. Liu, D. Zhu, S. P. Rodrigues, K. T. Lee, W. Cai Generative model for the inverse design of metasurfaces. *Nano Lett.* **18**, 6570–6576 (2018).

29. L. Gao, X. Li, D. Liu, L. Wang, Z. Yu A bidirectional deep neural network for accurate silicon color design. *Adv. Mater*. **31**, 1905467 (2019).

30. G. Wetzstein, A. Ozcan, S. Gigan, S. Fan, D. Englund, M. Soljačić, C. Denz, D. A. B. Miller, D. Psaltis Inference in artificial intelligence with deep optics and photonics. *Nature* **588**, 39–47 (2020).

31. Y. LeCun, Y. Bengio, G. Hinton, Deep learning. *Nature* **521**, 436–444 (2015).

32. R. Iten, T. Metger, H. Wilming, L. del Rio, R. Renner Discovering physical concepts with neural networks. *Phys. Rev. Lett.* **124**, 010508 (2020).

33. W. Ma, Z. Liu, Z. A. Kudyshev, A. Boltasseva, W. Cai, Y. Liu, Deep learning for the design of photonic structures. *Nat. Photon* **15**, 77–90 (2021).

34. C. Qian, B. Zheng, Y. Shen, L. Jing, E. Li, L. Shen, H. Chen Deep-learning-enabled self-adaptive microwave cloak without human intervention. *Nat. Photon* **14**, 383–390 (2020).

35. T. Yin, Z. Wei, X. Chen, Non-iterative methods based on singular value decomposition for inverse scattering problems. *IEEE Trans. Antennas Propag.* **68**, 4764–4773 (2020).

36. L. Mohjazi, A. Zoha, L. Bariah, S. Muhaidat, P. C. Sofotasios, M. A. Imran, O. A. Dobre, An outlook on the interplay of artificial intelligence and software-defined metasurfaces: An overview of opportunities and limitations. *IEEE Veh. Technol. Mag.* **15**, 62–73 (2020).

37. T. Cai, S. W. Tang, G. M. Wang, H. X. Xu, S. L. Sun, Q. He, L. Zhou High-performance bifunctional metasurfaces in transmission and reflection geometries. *Adv. Opt. Mater.* **5**, 1600506 (2017).

38. M. Jia, Z. Wang, H. Li, X. Wang, W. Luo, S. Sun, Y. Zhang, Q. He, L. Zhou Efficient manipulations of circularly polarized terahertz waves with transmissive metasurfaces. *Light Sci. Appl.* **8**, 16 (2019).

39. J. Peurifoy, Y. Shen, L. Jing, Y. Yang, F. C.-Renteria, B. G. De Lacy, J. D. Joannopoulos, M. Tegmark, M. Soljačić, Nanophotonic particle simulation and inverse design using artificial neural networks. *Sci. Adv.* **4**, eaar4206 (2018).

40. J. Jiang, J. A. Fan, Global optimization of dielectric metasurfaces using a physics-driven neural network. *Nano Lett.* **19**, 5366–5372 (2019).

41. A. Zhan, R. Gibson, J. Whitehead, E. Smith, J. R. Hendrickson, A. Majumdar, Controlling three-dimensional optical fields via inverse Mie scattering. *Sci. Adv.* **5**, eaax4769 (2019).

42. S. Molesky, Z. Lin, A. Y. Piggott, W. Jin, J. Vucković, A. W. Rodriguez Inverse design in nanophotonics. *Nat. Photon*. **12**, 659–670 (2018).

43. T. Li, A. Chen, L. Fan, M. Zheng, J. Wang, G. Lu, M. Zhao, X. Cheng, W. Li, X. Liu, H. Yin, L. Shi, J. Zi Photonic-dispersion neural networks for inverse scattering problems. *Light Sci. Appl.* **10**, 154 (2021).

44. T. Shan, X. Pan, M. Li, S. Xu, F. Yang Coding programmable metasurfaces based on deep learning techniques. *IEEE J. Emerg. Sel. Top Circuits Syst*. **10**, 114–125 (2020).

45. K. Simonyan A. Zisserman, Very deep convolutional networks for large-scale image recognition. arXiv:1409.1556 [cs.CV] (2015).

46. Z. Zhang, M. R. Sabuncu, Generalized cross entropy loss for training deep neural networks with noisy labels. arXiv:1805.07836 [cs.LG] (2018).

47. Z. Wang, C. Qian, T. Cai, L. Tian, Z. Fan, J. Liu, Y. Shen, L. Jing, J. Jin, E. P. Li, B. Zheng, H. Chen Demonstration of spider-eyes-like intelligent antennas for dynamically perceiving incoming waves. *Adv. Intell. Syst.* **3**, 2100066–8 (2021).

48. J. Chen, X. Chen, C. J. Liu, K. Huang, X. B. Xu Analysis of temperature effect on p-i-n diode circuits by a multiphysics and circuit cosimulation algorithm. *IEEE Trans Electron Devices* **59**, 3069–3077 (2012).

49. Q. Wang, E. Plum, Q. Yang, X. Zhang, Q. Xu, Y. Xu, J. Han, W. Zhang Reflective chiral meta-holography: Multiplexing holograms for circularly polarized waves. *Light Sci. Appl.* **7**, 25 (2018).

50. Z. Li, X. Tian, C. W. Qiu, J. S. Ho Metasurfaces for bioelectronics and healthcare. *Nat. Electron.* **4**, 382–391 (2021).

51. Q. Wang, E. T. F. Rogers, B. Gholipour, C. M. Wang, G. Yuan, J. Teng, N. I. Zheludev Optically reconfigurable metasurfaces and photonic devices based on phase change materials. *Nat. Photon* **10**, 60–65 (2016).

52. R. Zhu, T. Qiu, J. Wang, S. Sui, C. Hao, T. Liu, Y. Li, M. Feng, A. Zhang, C. W. Qiu, S. Qu Phase-to-pattern inverse design paradigm for fast realization of functional metasurfaces via transfer learning. *Nat. Commun.* **12**, 2974 (2021).

53. K. Chaudhuri, R. Salakhutdinov, *Online meta-learning*, paper presented at th Proceedings of the 36th International Conference on Machine Learning, California, 9 to 15 June 2019.

54. Y. Jia, C. Qian, Z. Fan, Y. Ding, Z. Wang, D. Wang, E.-P. Li, B. Zheng, T. Cai, H. Chen, In situ customized illusion enabled by global metasurface reconstruction. *Adv. Funct. Mater*. **32**, 2109331 (2022).

55. C. Balanis, *Antenna Theory* (Wiley, 2016).

56. H. Taghvaee, A. Cabellos-Aparicio, J. Georgiou, S. Abadal Error analysis of programmable metasurfaces for beam steering. *IEEE J. Emerg. Sel. Topics Power Electron.* **10**, 62–74 (2020).

57. J. Long, E. Shelhamer, T. Darrell, Fully convolutional networks for semantic segmentation. arXiv:1411.4038 [cs.CV] (2015).

58. D. Kingma, J. Ba, Adam: A method for stochastic optimization. arXiv*:*1412.6980 [cs.LG] (2014).

59. X. Glorot, Y. Bengio, Understanding the difficulty of training deep feedforward neural networks. *J. Mach. Learn Res*. **9**, 249–256 (2010).

60. K. Roth, H. Pirzadeh, A. L. Swindlehurst, J. A. Nossek A comparison of hybrid beamforming

and digital beamforming with low-resolution ADCs for multiple users and imperfect CSI. *IEEE J. Sel. Top. Signal Process*. **12**, 484–498 (2018).

61. A. M. Niknejad, D. Chowdhury, J. Chen, Design of CMOS power amplifiers. *IEEE Trans. Microw. Theory Tech.* **60**, 1784–1796 (2012).

62. W. Luo, S. Sun, H. Xu, Q. He, L. Zhou, Transmissive ultrathin Pancharatnam-Berry metasurfaces with nearly 100% efficiency. *Phys. Rev. Applied* **7**, 044033 (2017).

63. B. Xiong, L. Deng, R. Peng, Y. Liu Controlling the degrees of freedom in metasurface designs for multi-functional optical devices. *Nanoscale. Adv.* **1**, 3786–3806 (2019).

64. S. Sun, Q. He, S. Xiao, Q. Xu, X. Li, L. Zhou Gradient-index meta-surfaces as a bridge linking propagating waves and surface waves. *Nat. Mat.* **11**, 426–431 (2012).
